# Seroprevalence of antibody to influenza A(H1N1)pdm09 attributed to vaccination or infection, before and after the second (2010) pandemic wave in Australia

**DOI:** 10.1111/irv.12225

**Published:** 2013-12-31

**Authors:** Jodie McVernon, Karen Laurie, Helen Faddy, David Irving, Terry Nolan, Ian Barr, Anne Kelso

**Affiliations:** a Vaccine & Immunisation Research Group, Murdoch Children's Research Institute, Melbourne School of Population and Global Health, the University of MelbourneParkville, Vic., Australia; b Victorian Infectious Diseases Reference LaboratoryNorth Melbourne, Vic., Australia; c World Health Organisation Collaborating Centre for Reference and Research on InfluenzaNorth Melbourne, Vic., Australia; d Research and Development, Australian Red Cross Blood ServiceSydney, NSW, Australia

**Keywords:** Blood donors, immunity, herd, influenza, human, pandemics, serology, vaccination

## Abstract

**Objectives:**

Historical records of influenza pandemics demonstrate variability in incidence and severity between waves. The influenza A(H1N1)pdm09 pandemic was the first in which many countries implemented strain-specific vaccination to mitigate subsequent seasons. Serosurveys provide opportunity to examine the constraining influence of antibody on population disease experience.

**Design:**

Changes in the proportion of adults seropositive to influenza A(H1N1)pdm09over the 2009/10 (summer) interepidemic period and 2010 (winter) influenza season were measured to determine whether there was a temporal relationship with vaccine distribution and influenza activity, respectively.

**Setting:**

Australia.

**Sample:**

Plasma samples were collected from healthy blood donors from seven cities at the end of the first wave (November 2009), and before (March/April 2010) and after (November 2010) the subsequent influenza season.

**Main outcome measures:**

Haemagglutination inhibition (HI) assays were performed to assess reactivity of plasma against A(H1N1)pdm09, and the proportion seropositive (HI titre ≥ 40) compared over time, by age group and location.

**Results:**

Between the 2009 and 2010 influenza seasons, the seropositive proportion rose from 22% to 43%, an increase observed across all ages and sites. Brisbane alone recorded a significant rise in seropositivity over the 2010 influenza season – from a baseline of 35% to 53%. The seropositive proportion elsewhere was ≥40% pre-season, and did not rise over winter.

**Conclusions:**

A vaccine-associated increase in seropositive proportion preceding the influenza season correlated with low levels of disease activity in winter 2010. These observations support the role of immunisation in mitigating the ‘second wave’ of A(H1N1)pdm09, with timing critical to ensure sustained herd protection.

## Background and objectives

Epidemiologic records describing influenza activity over more than a century reveal the considerable challenges associated with predicting the behaviour of this virus in human populations. Recorded pandemics have shown marked variability in their extent and severity, associated with time, geographical location and population characteristics.^1^ While disease burden due to a novel strain is generally anticipated to decline over subsequent seasons as the population acquires immunity, morbidity and mortality have on occasion been observed to be higher in the ‘second wave’ than in the first, for reasons that remain unclear.^2^

The swine-origin influenza A(H1N1)pdm09 strain responsible for our most recent pandemic emerged in North America in March 2009 at a time of unprecedented global preparedness for such an event.^3^ Manufacture and widespread distribution of strain-specific vaccines was a key component of most preparedness and response strategies, although public acceptance of this intervention varied greatly by country and risk group.^4^ The 2009 pandemic is therefore the first in history to have been so extensively mitigated by vaccination, albeit delivered mostly within or following the first wave of infection.^3^ In Australia, an unadjuvanted monovalent A(H1N1)pdm09 vaccine (Panvax; CSL Ltd., Parkville, Australia) was made available for adults free of charge from 30 September 2009,^5^ more than 2 months after the peak of notifications, with a corresponding paediatric programme commencing in early December 2009.^6^

**Figure 1:**
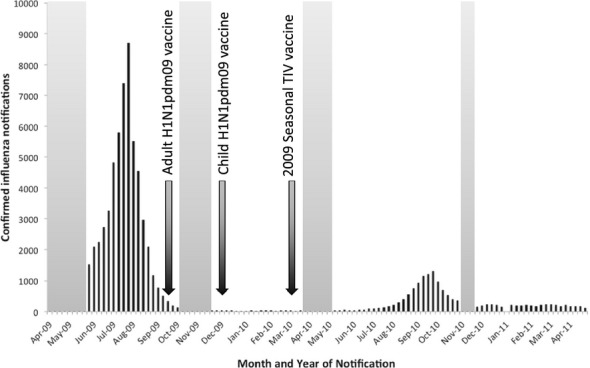
National confirmed influenza notifications by month, Australia, April 2009–May 2011 (source: National Notifiable Disease Surveillance System). The time series of national counts of laboratory-confirmed influenza notifications is shown in black. Shaded grey columns indicate windows of serosurvey specimen collection. Black arrows denote commencement of monovalent and seasonal influenza vaccine programmes.

Serosurveys conducted in several countries to date afford the opportunity to examine whether natural- and/or vaccine-induced antibody constrained the second (or in some cases third) pandemic wave.^7^–^12^ We here report on a study utilising plasma samples from healthy adult blood donors from selected large Australian cities that commenced in 2009.^13^ Ongoing specimen collections spanned the post-pandemic 2009/10 Southern Hemisphere summer during which a strain-specific vaccine was administered, and the subsequent 2010 winter influenza season, in which 64% of test-positive influenza specimens were attributed to A(H1N1)pdm09^14^ (Figure 1). Samples were tested for haemagglutination inhibition (HI) antibody specific to this novel strain, to determine whether there were changes in the seropositive proportion by age and location, in temporal association with vaccine distribution and influenza notifications in the general population. The ecological relationship between pre-season antibody titres and disease experience was also considered.

## Patients/methods

### Blood donor samples

**Figure 2:**
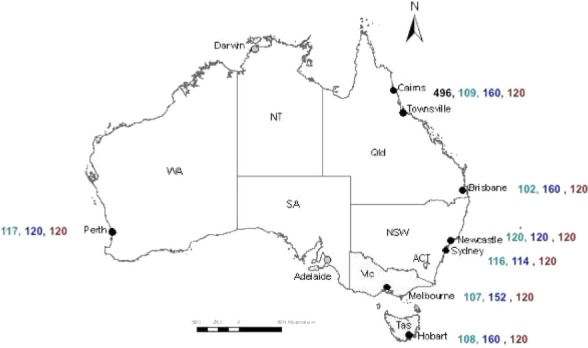
Geographical distribution of cities included in the study. Colours indicate the timing of sample collection: numbers of samples by site are shown pre-first wave (April/May 2009) in black, post-first wave (November 2009) in green, pre-second wave (March/April 2010) in blue and post-second wave (November 2010) in brown.

Plasma samples remaining after routine serology testing were prospectively collected from healthy adult Australian Red Cross Blood Service (Blood Service) donors attending collection centres in seven major cities around the country (Figure 2). Samples were collected at the end of the first pandemic wave (November 2009), prior to the 2010 influenza season (March/April 2010) and following the second wave (November 2010). Between 15 and 20 samples were collected at each timepoint in each city within the following age strata (years): 16–24, 25–34, 35–44, 45–54, 55–64 and ≥65 (i.e. 90–120 specimens at each site). In March/April 2010, additional specimens were collected from selected cities in the 16- to 24- and ≥65-year age groups (target of 40 in each) as these categories were of particular epidemiologic interest.

As previously described,^13^ a baseline collection of 500 randomly selected specimens, collected in Cairns/Townsville in April/May 2009 for dengue surveillance studies, was provided by the Blood Service for the assessment of seroprevalence of cross-reactive antibody prior to established community transmission of the A(H1N1)pdm09 strain.

This study was carried out under approval from the Blood Service Human Research Ethics Committee.

### Laboratory assays

Haemagglutination inhibition assays were performed at the World Health Organisation Collaborating Centre for Reference and Research on Influenza in Melbourne, Australia (WHO CC), to assess the reactivity of plasma against A(H1N1)pdm09. An egg-grown A/California/7/2009 reassortant virus was purified by sucrose gradient, concentrated and inactivated with β-propiolactone to create an influenza zonal pool preparation (gift from CSL Ltd). Plasma samples were pre-treated with receptor-destroying enzyme II (Denka Seiken Co. Ltd., Tokyo, Japan), 1:5 (volume/volume), and tested as previously described.^15^ Following a 1-hour incubation, 25 μl 1% (volume/volume) turkey red blood cells (RBC) was added to each well. Haemagglutination inhibition was read after 30 minutes. Any samples that bound to the RBC in the absence of virus were adsorbed with RBC for 1 hour and re-assayed. Titres were expressed as the reciprocal of the highest dilution of plasma where haemagglutination was prevented.

A panel of control samples was included in all assays, comprising paired sera from ferrets collected prior to and following infection with each of the following viruses: A(H1N1)pdm09, pre-pandemic influenza A(H1N1), A(H3N2) and influenza B. The panel further included human serum and plasma samples collected from individuals before April 2009, after known infection with the pandemic virus and following immunisation with the Australian monovalent A(H1N1)pdm09 vaccine. The threshold titre by which seropositivity was defined was the putative protective correlate of 40.^16^

### Statistical analysis and sample size

The proportion of donors seropositive was reported by age group and location for each timepoint, with 95% confidence intervals (95% CI), and compared over time for evidence of significant change using two-sample tests of proportion.

At each timepoint, there were approximately 140 individuals per group nationally collated by age stratum and 120 at each location. Assuming a starting seropositive proportion in the order of 20% at the end of the 2009 winter,^13^ a group size of 120–140 was required to report an increase in this proportion of 20%, with 90–94% power and 95% confidence, with only 37–41% power to detect a smaller increase of 10%. Collating all specimens at each timepoint, the minimum total of 800 samples provided 99% power to detect a 10% rise in seropositives, and 62% power to demonstrate a 5% rise, with 95% confidence.

## Results

### Study population

The number of samples collected from each study site at each timepoint is shown in Figure 2. A detailed age breakdown of participants by time and location of collection is provided in Table S1. In some instances, background reactivity could not be ameliorated by RBC adsorption, accounting for missing data on some participants when comparing these numbers with reported assay results in Table 1 and Table S2.

**Table 1:** Seropositive proportion by age group, before and after first and second pandemic waves

Age group (years)	Number of seropositive samples/total samples per group [Proportion (95% CI)]			
	Pre-first wave April/May 2009	Post-first wave November 2009	Pre-second wave March/April 2010	Post-second wave November 2010
16–24	14/74 0·19 (0·11, 0·30)	51/138 0·40 (0·29, 0·46)**	110/225 0·49 (0·42, 0·56) *	71/140 0·51 (0·42, 0·59)
25–34	4/59 0·07 (0·02, 0·16)	31/139 0·22 (0·16, 0·30)**	56/136 0·41 (0·33, 0·50)**	53/140 0·38 (0·30, 0·46)
35–44	8/64 0·13 (0·06, 0·23)	20/131 0·15 (0·09, 0·23)	45/139 0·32 (0·25, 0·41) *	61/140 0·44 (0·35, 0·52) *
45–54	12/129 0·09 (0·05, 0·16)	22/138 0·16 (0·10, 0·23)	45/140 0·32 (0·25, 0·41)**	52/139 0·37 (0·29, 0·46)
55–64	16/129 0·12 (0·07, 0·20)	26/131 0·20 (0·13, 0·28)	67/148 0·45 (0·37, 0·54)**	58/140 0·41 (0·33, 0·50)
>65	5/27 0·19 (0·06, 0·38)	25/102 0·25 (0·17, 0·34)	104/198 0·53 (0·45, 0·60) **	56/140 0·40 (0·32, 0·49) *
Total	59/496 0·12 (0·09, 0·15)	175/779 0·22 (0·20, 0·26) **	427/986 0·43 (0·40, 0·46) **	351/839 0·42 (0·38, 0·45)

* Significant change from previous timepoint, *P* ≤ 0·05.

** Significant change from previous timepoint, *P* < 0·005.

### Assay results

**Figure 3:**
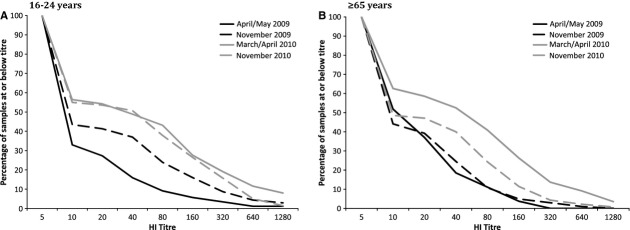
Reverse cumulative distribution plots of haemagglutination inhibition (HI) titres for two age groups (16–24 years and >65 years) across the four sampling timepoints. Cumulative proportion of specimens at each timepoint with titres at or below (on the premise that 20 < 40 and so on) a given dilution threshold, for participants aged (A) 16–24 years and (B) ≥65 years.

As previously demonstrated,^13^ adults ≥65 years of age had the highest levels of baseline cross-reactive antibody to A(H1N1)pdm09 prior to the first pandemic wave, with no detectable change over the 2009 influenza season. Significant increases in the proportion seropositive over the 2009 winter were noted in adults aged 16–24 and 25–34 years (Table 1, Figure 3).

Between the 2009 and 2010 influenza seasons, the proportion seropositive nationally rose by 21% from 22% to 43%, increasing significantly across all age groups (Table 1). Only adults aged 35–44 years exhibited a further (12%) rise in seropositivity during the 2010 winter, whereas a significant (13%) decline was observed among ≥65-year-olds over the same period (Table 1, Figure 3).

**Figure 4:**
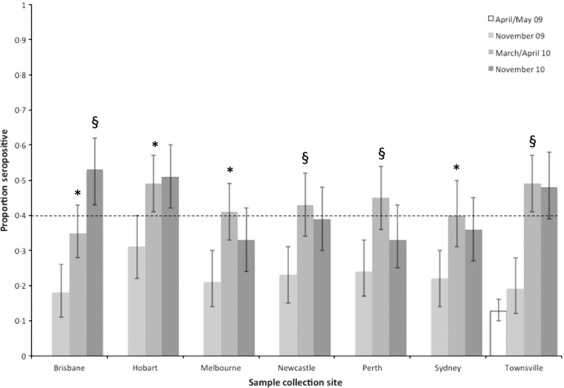
Seropositive proportion over time by city, with 95% binomial confidence intervals. Significant changes in proportion from the previous timepoint are denoted *P < 0·05; §*P* < 0·005 (see also Table S2). The dotted line indicates the 40% seropositive proportion, achieved by all cities but Brisbane prior to the 2010 winter. Note: pre-pandemic specimens were only available from the Townsville collection centre.

The proportion of donors seropositive was similar across all jurisdictions at the end of the first wave, and titres rose further over the 2009/10 (summer) interepidemic period, to varying degrees (Figure 4, Table S2). Notably, Brisbane was the only site to record a significant rise in seroprevalence over the following winter – from a pre-season baseline of 35% to 53% by November 2010 (Figure 4, Table S2). The seropositive proportion at all other sites was at least 40% prior to the 2010 influenza season and had not changed following the period of documented virus circulation.

## Conclusions

We observed a significant (21%) increase in the proportion of the Australian population seropositive to A(H1N1)pdm09 over the 2009/10 Southern Hemisphere summer, distributed across all age groups and jurisdictions (Table 1, Figure 4). This rise was temporally associated with a widely accessible government-funded monovalent pandemic vaccine programme. A telephone survey conducted by the Australian Institute of Health and Welfare between 11 January and 28 February 2010 reported adult (>18 years) pandemic vaccine uptake of 21%.^17^ This figure showed some non-significant variation with survey week, suggesting that the majority of vaccine had been received by early January 2010, and the only state to vary significantly from the national estimate was Tasmania (23·5%).^17^ Coverage increased by age, from those aged <20 years (<10%), through 20–54 years (13–16%) and 55–64 years (24%), to a maximum of 45% among those 65 years and above.^17^

Over the subsequent 2010 influenza season, a further increase in seroprevalence was observed in one city (Brisbane), the only site to report a pre-season seropositive proportion of less than 40% (Figure 4, Table S2). Available notifications data do not indicate an excess of cases in Queensland compared with other States and Territories, but differences in systems between jurisdictions make direct comparisons problematic.^18^ Elsewhere, the proportion seropositive did not change, with the exception of the elderly in whom antibodies appeared to wane. These findings are consistent with reports that influenza activity was generally low over the period (Figure 1).^18^

This study has a number of limitations that must be considered when interpreting its findings. The collections were cross-sectional in nature, precluding the assessment of rising or waning titres within individuals that would contribute to overall changes in the proportion seropositive over time. Utilisation of convenience specimens from healthy blood donors limited the age of participants to 16 years and over. Donors might differ from the general population in relation to illness avoidance behaviours (including attitudes to vaccination) as well as the prevalence of risk factors for infection. Accompanying information was restricted to age, sex and date of collection. Vaccination and prior influenza-like illness status were unknown, limiting inference to ecological association.

The laboratory methods used in this study were designed to minimise the higher background reactivity anticipated in plasma specimens compared with serum. Despite efforts at standardisation, HI assay results differ significantly between laboratories.^19^ Prior immunisation with inactivated influenza vaccines has been demonstrated to blunt the antibody response to virologically confirmed infection,^20^ potentially biasing inferences of population infection exposure based on changes in the seropositive proportion over the second wave. Interpretation of HI assay findings is further complicated by the absence of definitive correlates of protection against infection, reflecting the fact that such assays consider only one aspect of immunity to influenza.^16^

Despite these limitations, comparison of our results with other recently published serosurveys demonstrates interesting differences and similarities in the international experience of subsequent pandemic waves. In England, monovalent pandemic vaccines were available for adults from the early phase of the second pandemic wave (August 2009) and for children from the end of the second pandemic wave (January 2010).^7^ Haemagglutination inhibition assays conducted on English residual diagnostic sera revealed an increase in the proportion of seropositive (HI titre ≥32) 0- to 5-year-olds over the months between the end of the second wave and onset of the third that was likely attributable to vaccination, with no change or a decline in all other age groups.^7^ During the third wave, significant rises in seropositivity (20–35%) occurred primarily in younger adults, consistent with an upward age shift in reported disease during a substantial epidemic season. A similar increase in seropositive proportion was observed among the elderly, associated with high coverage of the A(H1N1)pdm09-containing 2010/11 seasonal trivalent vaccine, rather than disease.^7^ Relative sparing of children in the third wave in comparison with earlier pandemic waves was attributed to the recency of the paediatric-targeted vaccine campaign.^7^ Similar findings were observed in a Scottish study.^11^

In Sweden, a monovalent AS03-adjuvanted pandemic vaccine was made freely available from October 2009, coinciding with onset of widespread disease activity that ultimately peaked in November.^12^ Vaccine continued to be distributed until March 2010, by which time population uptake was estimated to be 60% across all age groups.^12^ Almost half of the population was seropositive by May 2010, although the relative fraction attributable to vaccination or exposure was unclear.^12^ By May 2011, and in contrast to the UK experience, seroprevalence remained high, with significant increases in 2- to 4-year-olds and 15- to 24-year-olds believed to be due to mild unrecognised infection, as reported disease activity in the 2010/11 season was low.^12^ Corresponding rises in the seropositive proportion in those aged 65 years and over were ascribed to A(H1N1)pdm09-containing seasonal vaccine coverage in excess of 50%.^12^

Large prospective studies conducted in southern^9^ and northern^8^ China over 2010/11 shed further light on the relationship between immunity and disease, in a year in which both tropical and temperate regions reported low levels of A(H1N1)pdm09 activity between late December 2010 and early February 2011.^21^ China was the first country to report the development of a monovalent pandemic vaccine,^22^ which was widely distributed from October 2009 onwards over an extended period.^9^

A three-timepoint collection from Guangdong spanning bimodal summer and winter influenza seasons demonstrated a significantly higher (˜30%) seropositive proportion among vaccinated than among unvaccinated participants within each sampling window.^22^ Seroprevalence in unvaccinated participants declined within months of cessation of the first pandemic wave, rising significantly (by ˜4%) over the second wave, with opposite trends of equivalent magnitude observed in the vaccinated group, demonstrating heterogeneity of population experience associated with immunisation status.^22^

More than 4500 serum samples were collected in Beijing in September 2010 and compared with a similar number from April 2011. Cross-sectional comparison revealed a small (3%) but significant rise in the seropositive proportion over the winter season, driven by disease in the very young (0–5 years) and vaccination in the elderly (>60 years).^8^ Of note, a nested longitudinal cohort involving a subset of 1217 participants demonstrated a seroconversion rate (fourfold titre rise) over the same period of 14·5%, without a change in cross-sectional seroprevalence.^9^ Seroconversion was significantly associated with receipt of the 2010/11 seasonal trivalent influenza vaccine, but only among individuals without prior immunity or a history of monovalent A(H1N1)pdm09 vaccine administration.^8^

The diverse population experiences described above provide strong suggestive evidence of the role of herd immunity in constraining subsequent pandemic waves. The simplest conceptualisation of the critical herd immunity threshold required for population protection against influenza derives from mathematical models assuming equal population mixing and susceptibility, upon which basis a value of 33% was proposed as sufficient in a recent German study.^23^ Methods that incorporate basic determinants of heterogeneity such as age clearly demonstrate the limitations of such assumptions, particularly when extrapolating vaccine coverage thresholds from one population to another with a different age-dependent risk profile.^24^,^25^

Whatever the situation-specific threshold may be, review of these serosurvey findings indicates that timing of vaccine delivery for mitigation of subsequent seasons should be carefully considered. Differences in the duration of protection offered by infection and vaccination will be highly influential in determining the population immune profile in subsequent seasons. In the absence of individual-level data from Australia, the UK and Sweden, it is hard to disentangle the relative importance of the timing and source of exposure (infection, adjuvanted versus unadjuvanted vaccine) as determinants of antibody persistence through to the ‘second wave’.

Even though our serosurvey had limitations, it has been valuable for within-country assessment of pandemic exposures and interventions,^13^ and together with similar studies conducted in other settings, it has usefully informed international comparisons of impact.^26^ Recognising the value of this surveillance platform, a number of influenza specialists from several international organisations, including the World Health Organisation, have assembled an international working group to improve harmonisation of serosurvey conduct and reporting, providing a more robust evidence base for international public health decision support in future pandemic events.^27^

## Supporting Information

**Table S1.** Number of specimens collected, by age and location, at each timepoint. **Table S2.** Proportion seropositive by location, at each timepoint.
