# Supplementary material for: Seroprevalence of antibody to influenza A(H1N1)pdm09 attributed to vaccination or infection, before and after the second (2010) pandemic wave in Australia
Source: Influenza Other Respir Viruses. 2013 Dec 31;8(2):194–200. doi: 10.1111/irv.12225 (PMC4186467; doi:10.1111/irv.12225)
Supplement: Supplementary file 1 [file irv0008-0194-SD1.docx]

**Supplementary Table 1: Number of specimens collected, by age and location, at each timepoint**

| Collection Site | Sample no (n) | 16-24 yrs | 25-34 yrs | 35-44 yrs | 45-54 yrs | 55-64 yrs | >65 yrs | % Male |
| --- | --- | --- | --- | --- | --- | --- | --- | --- |
| **APRIL/MAY 2009 BASELINE COLLECTION** | | | | | | | | |
| Cairns/Townsville  20^th^ April–9^th^ May | 501 | 88 | 59 | 64 | 129 | 132 | 29 | 54 |
| **OCTOBER/NOVEMBER 2009 COLLECTION** | | | | | | | | |
| Brisbane, 22-30^th^ Oct | 107 | 20 | 21 | 16 | 20 | 20 | 10 | 65 |
| Hobart, 16^th^ Nov-1^st^ Dec | 114 | 20 | 21 | 20 | 20 | 19 | 14 | 44 |
| Melbourne, 16^th^ Nov | 113 | 20 | 20 | 20 | 20 | 20 | 13 | 54 |
| Newcastle, 24-26^th^ Nov | 120 | 20 | 20 | 20 | 20 | 20 | 20 | 59 |
| Perth, 17-18^th^ Nov | 120 | 20 | 20 | 20 | 20 | 20 | 20 | 50 |
| Sydney, 19-20^th^ Nov | 120 | 20 | 20 | 20 | 20 | 20 | 20 | 65 |
| Townsville, 13-27^th^ Oct | 112 | 19 | 21 | 20 | 20 | 21 | 11 | 63 |
| **MARCH/APRIL 2010 COLLECTION** | | | | | | | | |
| Brisbane, 22^nd^-25^th^ March | 160 | 40 | 20 | 20 | 20 | 20 | 40 | 51 |
| Hobart, 23^rd^ March-6^th^ April | 160 | 40 | 20 | 20 | 20 | 21 | 39 | 52 |
| Melbourne, 23^rd^ March-1^st^ April | 152 | 42 | 19 | 19 | 20 | 26 | 26 | 54 |
| Newcastle, 24^th^-28^th^ April | 120 | 20 | 20 | 20 | 20 | 20 | 20 | 52 |
| Perth, 29^th^ April-5^th^ May | 120 | 23 | 17 | 20 | 20 | 22 | 18 | 51 |
| Sydney, 24^th^-30^th^ April | 114 | 20 | 20 | 20 | 20 | 19 | 15 | 50 |
| Townsville, 9^th^-18^th^ March | 160 | 40 | 20 | 20 | 20 | 20 | 40 | 56 |
| **NOVEMBER 2010 COLLECTION** | | | | | | | | |
| Brisbane, 19^th^-25^th^ November | 120 | 20 | 20 | 20 | 20 | 20 | 20 | 60 |
| Hobart, 10^th^-25^th^ November | 120 | 20 | 20 | 20 | 20 | 20 | 20 | 53 |
| Melbourne, 10^th^-12^th^ November | 120 | 20 | 20 | 20 | 20 | 20 | 20 | 47 |
| Newcastle, 10^th^-30^th^ November | 120 | 20 | 20 | 20 | 20 | 20 | 20 | 54 |
| Perth, 25^th^-29^th^ November | 120 | 20 | 20 | 20 | 19 | 21 | 20 | 53 |
| Sydney, 10^th^-30^th^ November | 120 | 20 | 20 | 20 | 20 | 20 | 20 | 54 |
| Townsville, 18^th^-25^th^ November | 120 | 20 | 20 | 20 | 20 | 20 | 20 | 68 |

**Supplementary Table 2: Proportion seropositive by location, at each timepoint**

| Collection Site | n/N seropositive | Proportion (95% CI) |
| --- | --- | --- |
| **APRIL/MAY 2009 BASELINE COLLECTION** | | |
| Cairns/Townsville | 59/496 | 0.12 (0.09, 0.15) |
| **NOVEMBER 2009 COLLECTION** | | |
| Brisbane | 18/102 | 0.18 (0.11, 0.26) |
| Hobart | 33/108 | 0.31 (0.22, 0.40) |
| Melbourne | 23/107 | 0.21 (0.14, 0.30) |
| Newcastle | 27/120 | 0.23 (0.15, 0.31) |
| Perth | 28/117 | 0.24 (0.17, 0.33) |
| Sydney | 25/116 | 0.22 (0.14, 0.30) |
| Townsville | 21/109 | 0.19 (0.12, 0.28) |
| All November 2009 | 175/779 | 0.22 (0.20, 0.26) |
| **MARCH/APRIL 2010 COLLECTION** | | |
| Brisbane | 56/160 | 0.35 (0.28, 0.43) * |
| Hobart | 79/160 | 0.49 (0.41, 0.57) * |
| Melbourne | 62/152 | 0.41 (0.33, 0.49) * |
| Newcastle | 51/120 | 0.43 (0.34, 0.52) § |
| Perth | 54/120 | 0.45 (0.36, 0.54) § |
| Sydney | 46/114 | 0.40 (0.31, 0.50) * |
| Townsville | 79/160 | 0.49 (0.41, 0.57) § |
| All March/April 2010 | 427/986 | 0.43 (0.40, 0.46) § |
| **NOVEMBER 2010 COLLECTION** | | |
| Brisbane | 63/120 | 0.53 (0.43, 0.62) § |
| Hobart | 61/120 | 0.51 (0.42, 0.60) |
| Melbourne | 39/120 | 0.33 (0.24, 0.42) |
| Newcastle | 47/120 | 0.39 (0.30, 0.48) |
| Perth | 40/120 | 0.33 (0.25, 0.43) |
| Sydney | 43/120 | 0.36 (0.27, 0.45) |
| Townsville | 58/120 | 0.48 (0.39, 0.58) |
| All November 2010 | 351/840 | 0.42 (0.38, 0.45) |

P values from two-sample test of proportions reported.

* Significant change from previous timepoint, p≤0.05

§ Significant change from previous timepoint, p<0.005
